# Technical challenges of quantitative chest MRI data analysis in a large cohort pediatric study

**DOI:** 10.1007/s00330-018-5863-7

**Published:** 2018-12-05

**Authors:** Anh H. Nguyen, Adria Perez-Rovira, Piotr A. Wielopolski, Juan A. Hernandez Tamames, Liesbeth Duijts, Marleen de Bruijne, Andrea Aliverti, Francesca Pennati, Tetyana Ivanovska, Harm A. W. M. Tiddens, Pierluigi Ciet

**Affiliations:** 1 Department of Pediatrics, Division of Respiratory Medicine and Allergology, Erasmus Medical Center, Wytemaweg 80, 3015 CN Rotterdam, the Netherlands; 2 Department of Radiology, Erasmus Medical Center, Wytemaweg 80, 3015 CN Rotterdam, the Netherlands; 3 Department of Medical Informatics, Erasmus Medical Center, Wytemaweg 80, 3015 CN Rotterdam, the Netherlands; 4 Department of Pediatrics, Division of Neonatology, Erasmus Medical Center, University Medical Center Rotterdam, Wytemaweg 80, 3015 CN Rotterdam, the Netherlands; 5 The Generation R Study Group, Erasmus Medical Center, University Medical Center Rotterdam, Wytemaweg 80, 3015 CN Rotterdam, the Netherlands; 6 Department of Computer Science, University of Copenhagen, Nørregade 10, 1165 Copenhagen, Denmark; 7 Department of Electronics, Information and Bioengineering, Polytechnic University of Milan, Piazza Leonardo da Vinci 32, 20133 Milan, Italy; 8 Department for Computational Neuroscience, Georg-August-Universität Göttingen, Friedrich-Hund Platz 1, 37077 Göttingen, Germany; 9 Department of Radiology and Pediatric Pulmonology, Erasmus Medical Center, Sophia Children’s Hospital, Wytemaweg 80, 3015 CN Rotterdam, the Netherlands

**Keywords:** Magnetic resonance imaging, Lung, Lung volume measurements, Phantoms, Imaging

## Abstract

**Objectives:**

This study was conducted in order to evaluate the effect of geometric distortion (GD) on MRI lung volume quantification and evaluate available manual, semi-automated, and fully automated methods for lung segmentation.

**Methods:**

A phantom was scanned with MRI and CT. GD was quantified as the difference in phantom’s volume between MRI and CT, with CT as gold standard. Dice scores were used to measure overlap in shapes. Furthermore, 11 subjects from a prospective population-based cohort study each underwent four chest MRI acquisitions. The resulting 44 MRI scans with 2D and 3D Gradwarp were used to test five segmentation methods. Intraclass correlation coefficient, Bland–Altman plots, Wilcoxon, Mann–Whitney *U*, and paired *t* tests were used for statistics.

**Results:**

Using phantoms, volume differences between CT and MRI varied according to MRI positions and 2D and 3D Gradwarp correction. With the phantom located at the isocenter, MRI overestimated the volume relative to CT by 5.56 ± 1.16 to 6.99 ± 0.22% with body and torso coils, respectively. Higher Dice scores and smaller intraobject differences were found for 3D Gradwarp MR images. In subjects, semi-automated and fully automated segmentation tools showed high agreement with manual segmentations (ICC = 0.971–0.993 for end-inspiratory scans; ICC = 0.992–0.995 for end-expiratory scans). Manual segmentation time per scan was approximately 3–4 h and 2–3 min for fully automated methods.

**Conclusions:**

Volume overestimation of MRI due to GD can be quantified. Semi-automated and fully automated segmentation methods allow accurate, reproducible, and fast lung volume quantification. Chest MRI can be a valid radiation-free imaging modality for lung segmentation and volume quantification in large cohort studies.

**Key Points:**

*• Geometric distortion varies according to MRI setting and patient positioning.*

*• Automated segmentation methods allow fast and accurate lung volume quantification.*

*• MRI is a valid radiation-free alternative to CT for quantitative data analysis.*

**Electronic supplementary material:**

The online version of this article (10.1007/s00330-018-5863-7) contains supplementary material, which is available to authorized users.

## Introduction

Computed tomography (CT) is the most used technique for quantitative lung imaging because of its high spatial resolution and signal-to-noise ratio [1, 2]. Consequently, quantitative imaging analysis using chest CT is better developed and validated compared to chest magnetic resonance imaging (MRI) [3–6]. Nevertheless, MRI is being developed as a feasible radiation-free alternative imaging modality [1, 7].

However, several technical challenges hamper quantitative analysis with MRI, namely protocol standardization, low signal-to-noise ratio, and low spatial resolution. Volume computation in MR is also limited due to geometric distortion (GD) [1, 3], which is mainly caused by magnetic field inhomogeneity and nonlinearity of gradient coils within the scanner [7, 8]. Image processing techniques are available for GD correction and are commonly employed by manufacturers (i.e., “Gradwarp,” General Electric Healthcare). These techniques are particularly important for the delineation of target volumes for radiotherapy of cancer, where several models to correct GD have been analyzed [8–13].

Existing literature has focused on data with relatively small field-of-view (FOV) or on anatomical locations close to the isocenter where GD is minimal, such as in MRI protocols for prostate, brain, and neck tumor size quantification [7, 14, 15]. Conversely, lung imaging requires larger FOV and is therefore more influenced by GD, as magnetic field inhomogeneities make GD more pronounced the farther the object scanned is from the isocenter [7, 10, 16]. Consequently, peripheral lung portions (i.e., costophrenic angles) are most affected by GD. In addition, different MRI settings and patient’s positioning can influence the magnitude of GD [7, 17].

To the best of our knowledge, no previous publications have assessed the effect of GD on lung volume quantification in chest MRI and specifically the validation of lung volume quantification in MRI against CT. This study addresses the problem of correcting for magnetic field inhomogeneity.

In addition, this study evaluates manual, semi-automated, and fully automated methods for lung segmentation and volume quantification with MRI. Lung segmentation is a fundamental step for image analysis and is aimed to extract quantitative information. Although manual segmentation with delineation of lung boundaries on each image can give accurate results, it is laborious. Therefore, various segmentation methods have been developed for CT images. Few studies have been conducted on segmentation methods for MR images [18, 19], because it is believed to be more difficult and to have more variations than CT volumetry. In this study, we assessed segmentation methods in accuracy, reproducibility, and time efficiency to determine the best segmentation strategy for lung volume quantification using MRI in the Generation R Study, a large prospective population-based cohort study, described in detail in the Supplementary material.

In summary, we aimed (1) to quantify GD for different MRI scan settings on volume measurements compared to CT using phantoms and (2) to assess the accuracy, reproducibility, and time efficiency of semi-automated and fully automated lung volume segmentation tools compared to manual segmentations of MRI measurements obtained in children.

## Materials and methods

### Datasets

#### Phantom data

A MRI body phantom (Fig. E1), with four bottles filled with potassium sorbate (General Electric Healthcare), was used to assess the effect of GD on volume quantification depending on six different scan settings: (1) reference position with phantom centered in the scanner isocenter, (2) electronic displacement of FOV to simulate incorrect FOV positioning by a MRI technician, (3) manual displacement of phantom to simulate possible patient’s movements in the scanner, (4) table repositioning to simulate whole-body MRI protocol, (5) parallel imaging with different acceleration factors for faster image acquisition, and (6) use of torso coil to replicate the lung MRI protocol of the prospective population-based Generation R cohort study. For settings (2) and (3), eight different phantom positions distanced 5 cm from the isocenter were tested: left (L), right (R), inferior (I), superior (S), left inferior (LI), right inferior (RI), left superior (LS), and right superior (RS). For setting (5), four different acceleration factors were tested (1, 2.25, 4, 5). For setting (6), three positions were tested: torso coil centered on the subject and torso coil distanced 5 cm left or right from the center. All images, except setting (6), were acquired with body coil to obtain the most homogenous signal from the phantom and thus facilitating volume segmentation. Images were collected with in-plane bidimensional (2D Gradwarp) and full three-dimensional (3D Gradwarp) GD correction. These correction techniques correct spatial distortion artifacts and blurring at the extreme margins of MR images determined by only nongradient field nonlinearity [20].

#### Subjects’ data

**Fig. 1:**
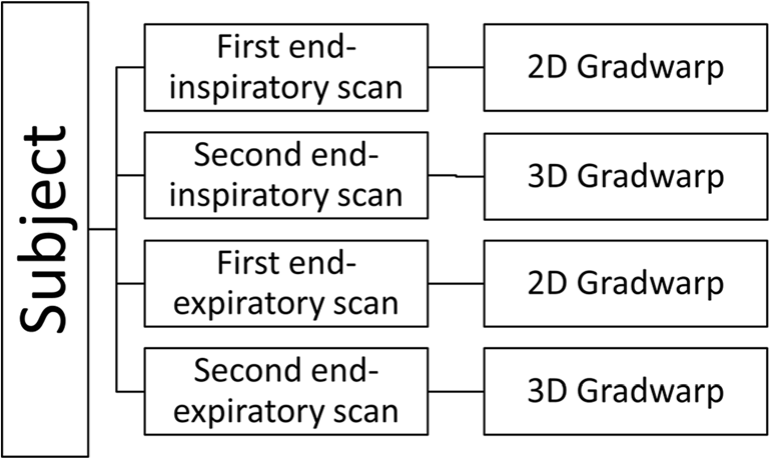
Flowchart of acquisition scheme per subject. Each subject (*n* = 11) underwent two end-inspiratory and two end-expiratory acquisitions. 2D and 3D Gradwarp correction was applied to one end-inspiratory and one end-expiratory scan. In total, 11 subjects underwent four acquisitions, resulting in 44 scans

To test lung volume segmentation methods, lung MRI data of a subset of 11 anonymized children were randomly selected from the Generation R Study [21, 22]. After written informed consent (METC-2012-165), children underwent whole-body MRI, including brain, heart, hips, and lung MRI acquisitions. The MRI scans were carried out in a specially designed child-friendly MRI research facility. From November 2014 to January 2016, 5000 MRI scans were acquired in the Generation R Study. Each subject underwent two end-inspiratory and two end-expiratory spirometer-guided MRI acquisitions. Data were acquired with 2D and 3D Gradwarp. In particular, 3D Gradwarp of the scanner was applied to one end-inspiratory and one end-expiratory scan (Fig. 1).

More information about the Generation R Study and parameters for MRI and CT acquisitions are presented in the Supplementary material*.*

### Imaging analysis

#### Phantom segmentation

**Fig. 2:**
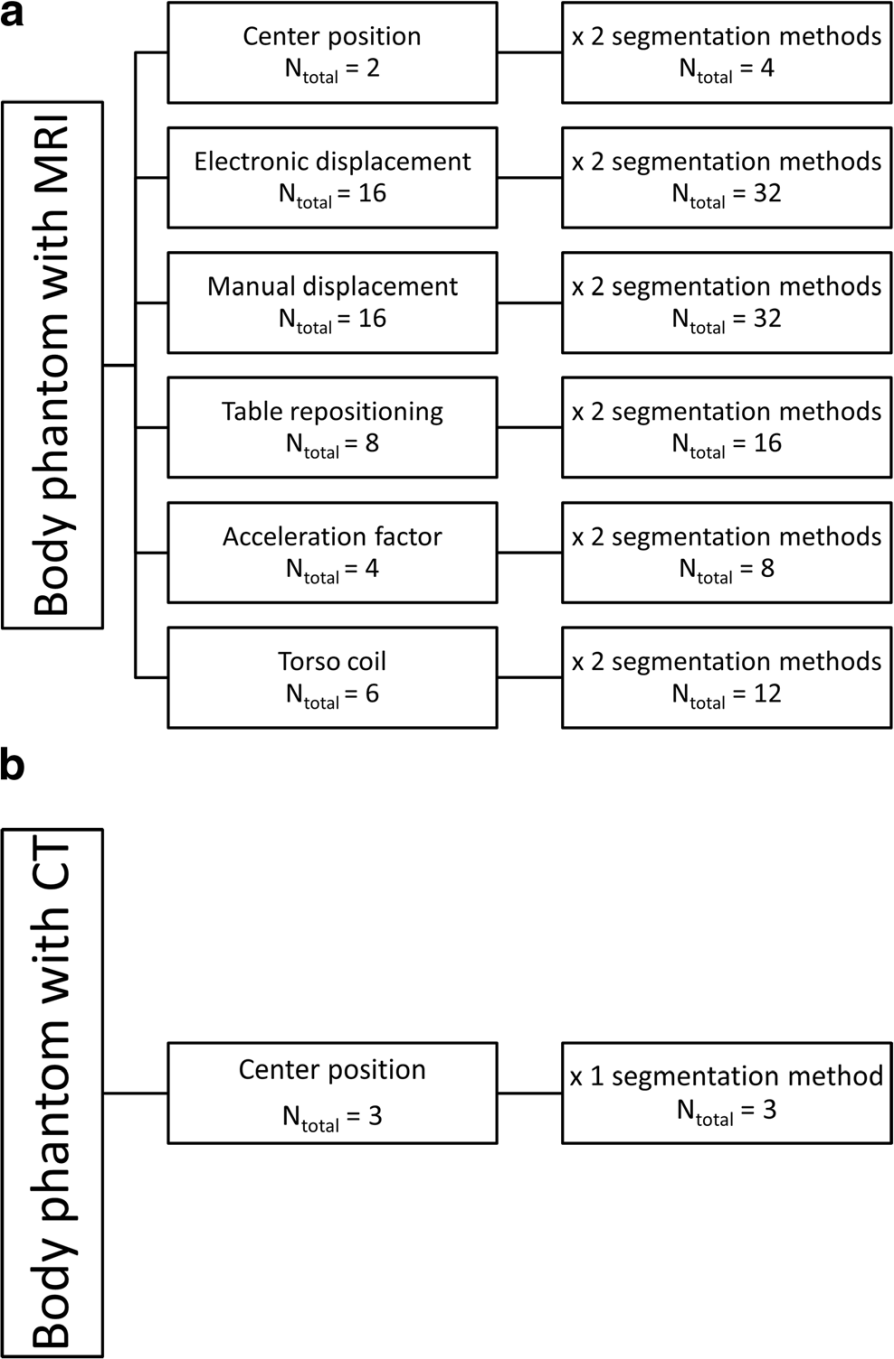
MRI and CT acquisition scheme of body phantom. **a** Phantom acquisitions with MRI with six different scan settings. **b** Phantom acquisitions with CT scan as reference images

Phantom volume measurements were manually obtained with MRI and CT through signal intensity thresholding segmentation using AW Server 2 platform (AWS) by GEHC and 3D Slicer software (http://www.slicer.org) by a single observer. Signal intensity threshold was chosen specifically for each scan to include the entire volume of interest, which was visually inspected in multiplanar reformats. All 52 MRI phantom acquisitions were segmented, once with AWS and once with 3D Slicer, making a total of 104 segmentations. Three CT phantom segmentations were performed with 3D Slicer (Fig. 2) to obtain true volumes. A total of 20 out of 52 acquisitions were randomly selected for second segmentation with AWS and 3D Slicer to assess intra- and intermethod agreement.

#### Subjects’ lung segmentation

Five lung segmentation methods were tested in 44 scans from 11 subjects (Fig. E2): one fully manual (ITK-SNAP) [23], two semi-automated (3D Slicer [24] and GeoS [25]), and two fully automated (Ivanovska [26] and Pennati [27]). Each segmentation method is explained in the Supplementary material.

Five out of 11 Generation R subjects, with four acquisitions for each subject, were randomly selected for second segmentation with manual and semi-automated methods by the first observer and a second observer to assess intra- and interobserver agreement. Both observers were blinded to each other’s segmentations.

#### Quantification of GD on phantom

MRI volume measurements were compared to CT measurements as the gold standard. The magnitude of GD was quantified as relative volume difference between MRI and CT measurements.

Phantom volume segmentations of the aforementioned MRI scan settings were compared to reference CT images using Dice score after rigid registration. Dice score measures volumetric overlap in the range between 0 (no overlap) to 1 (complete overlap) [28] and can be seen as a measure of shape similarity.

To assess intraobserver agreement, 20 randomly selected phantom acquisitions were segmented twice by the first observer.

#### Quantification of GD on patient’s data

Volume differences between 2D and 3D Gradwarp datasets were computed as volume difference between the two end-inspiratory scans. Inspiratory scans were used because of easier segmentation of the volume of interest due to more homogeneous lung parenchyma signal intensity levels. As the entire Generation R cohort was only acquired with 2D Gradwarp, 3D Gradwarp correction performed with the built-in software of the scanner (3D GW_scanner_) was compared with an offline software (3D GW_off-line_). Further details are provided in the online supplement.

#### Comparison of lung segmentation tools on subjects’ data

Lung segmentations from end-inspiratory scans were obtained using 3D Slicer (with threshold painting tool) [24], GeoS [25], Ivanovska [26, 29], and Pennati [27] methods and compared to manual segmentation (MS) to assess their performance. At the time of analysis, the tested fully automated methods were not able to perform segmentation of end-expiratory scans due to decreased contrast differences between lung parenchyma and surrounding tissues. Consequently, only 3D Slicer and GeoS methods were compared to MS for end-expiratory scans. Mean segmentation time for end-inspiratory and end-expiratory images was calculated for each method. Vital capacity (VC) computed with MRI (VC_MRI_) by segmentation was compared to VC measured by spirometry (VC_SPIROMETER_) to assess correlation. The smallest measured end-expiratory lung volume was subtracted from the largest measured end-inspiratory lung volume to compute VC_MRI_. VC_MRI_ was compared to the highest VC_SPIROMETER_. VC_SPIROMETER_ was obtained from spirometry. VC_MRI_ was calculated as the volume difference between inspiratory and expiratory levels.

### Statistical analysis

Descriptive data were reported as means ± standard deviations. *Q*–*Q* plots and Shapiro–Wilk tests were used to test normality. Intraclass correlation (ICC) coefficient and Bland–Altman plots were used to assess intra- and interobserver agreement. Paired samples *t* test, Wilcoxon signed-ranks test, or Mann–Whitney *U* test was applied to assess differences in lung volume measurements.

To compare semi-automated and fully automated segmentation methods with MS, volume differences were calculated as absolute and relative difference. Dice scores were used to measure overlap in shapes. Pearson correlation coefficient was used to determine correlation between VC_MRI_ and VC_SPIROMETER_. *P* values ≤ 0.05 were considered to be statistically significant. Multiple comparisons were adjusted using Bonferroni correction. Statistical analyses were performed using SPSS v.21 (IBM SPSS Statistics).

## Results

### Quantification of GD on phantom’s data

**Fig. 3:**
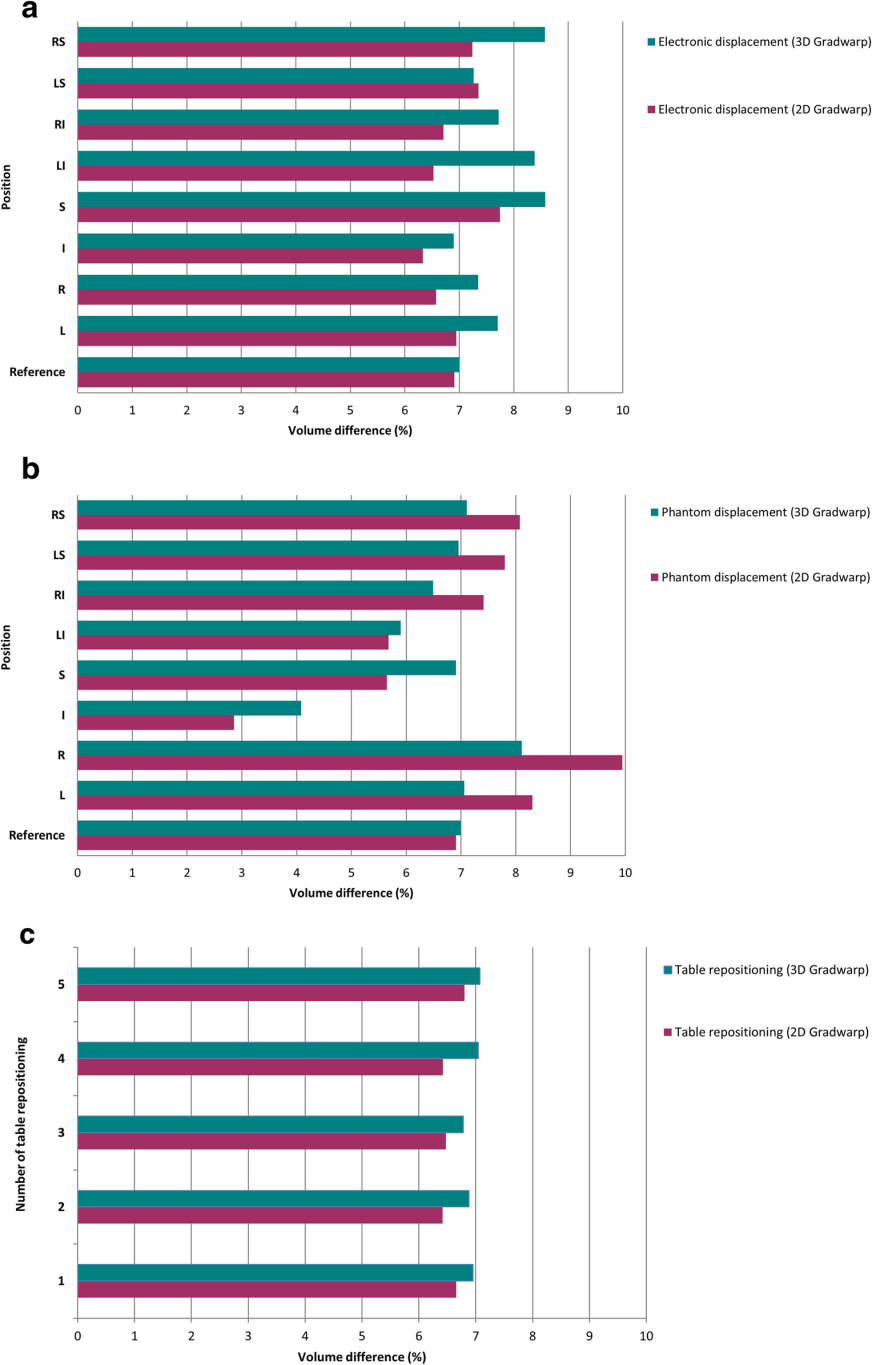

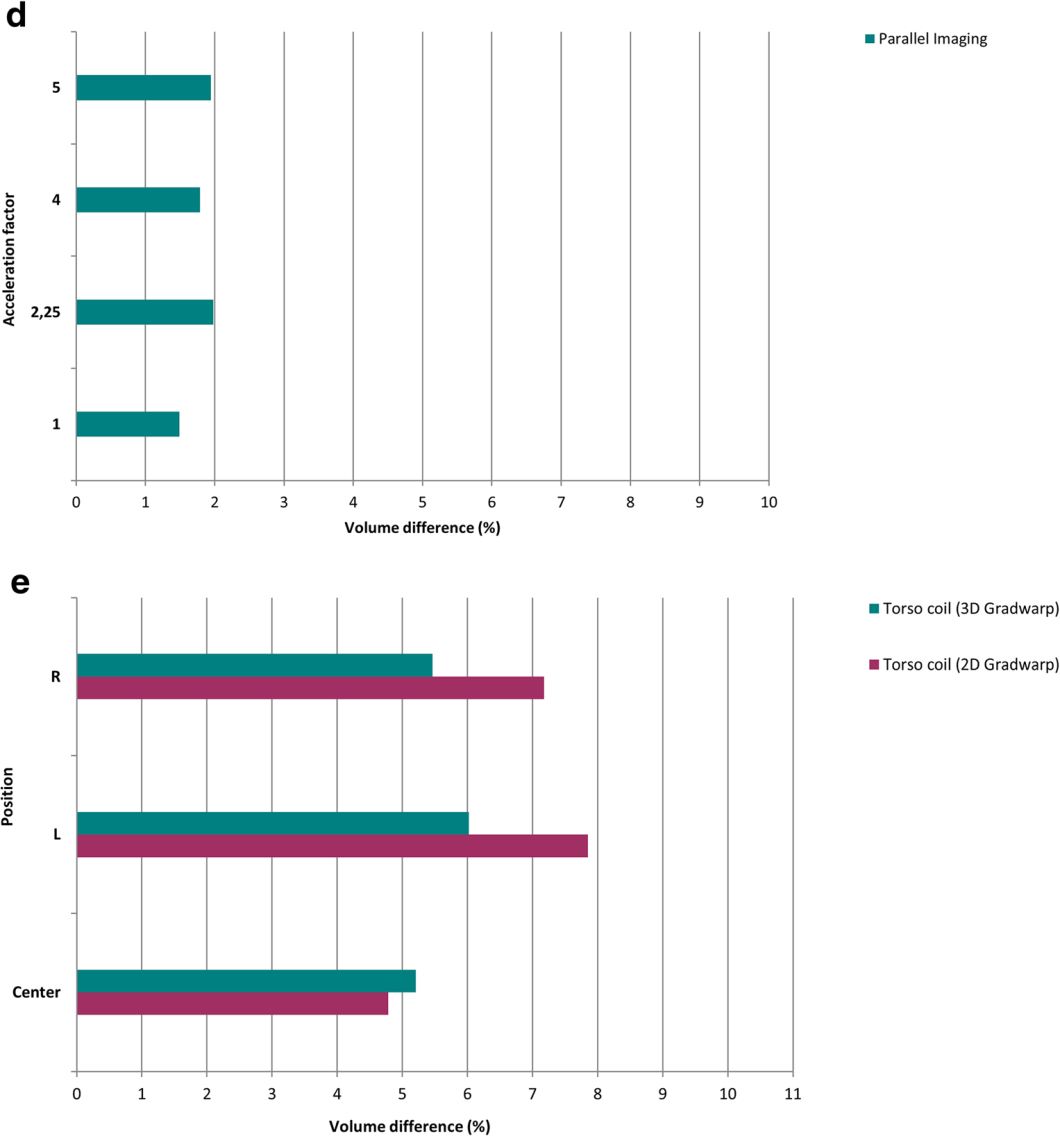
Effect of GD on volume quantification with MRI compared to CT according to various MRI scan settings. Relative volume difference (%) for **a** electronic displacement of FOV, **b** manual displacement of phantom, **c** table repositioning, **d** parallel imaging, and **e** use of torso coil. Reference = reference MRI isocenter position. Positions distanced 5 cm from isocenter: L = left, R = right, I = inferior, S = superior, LI = left inferior, RI = right inferior, LS = left superior, RS = right superior

**Fig. 4:**
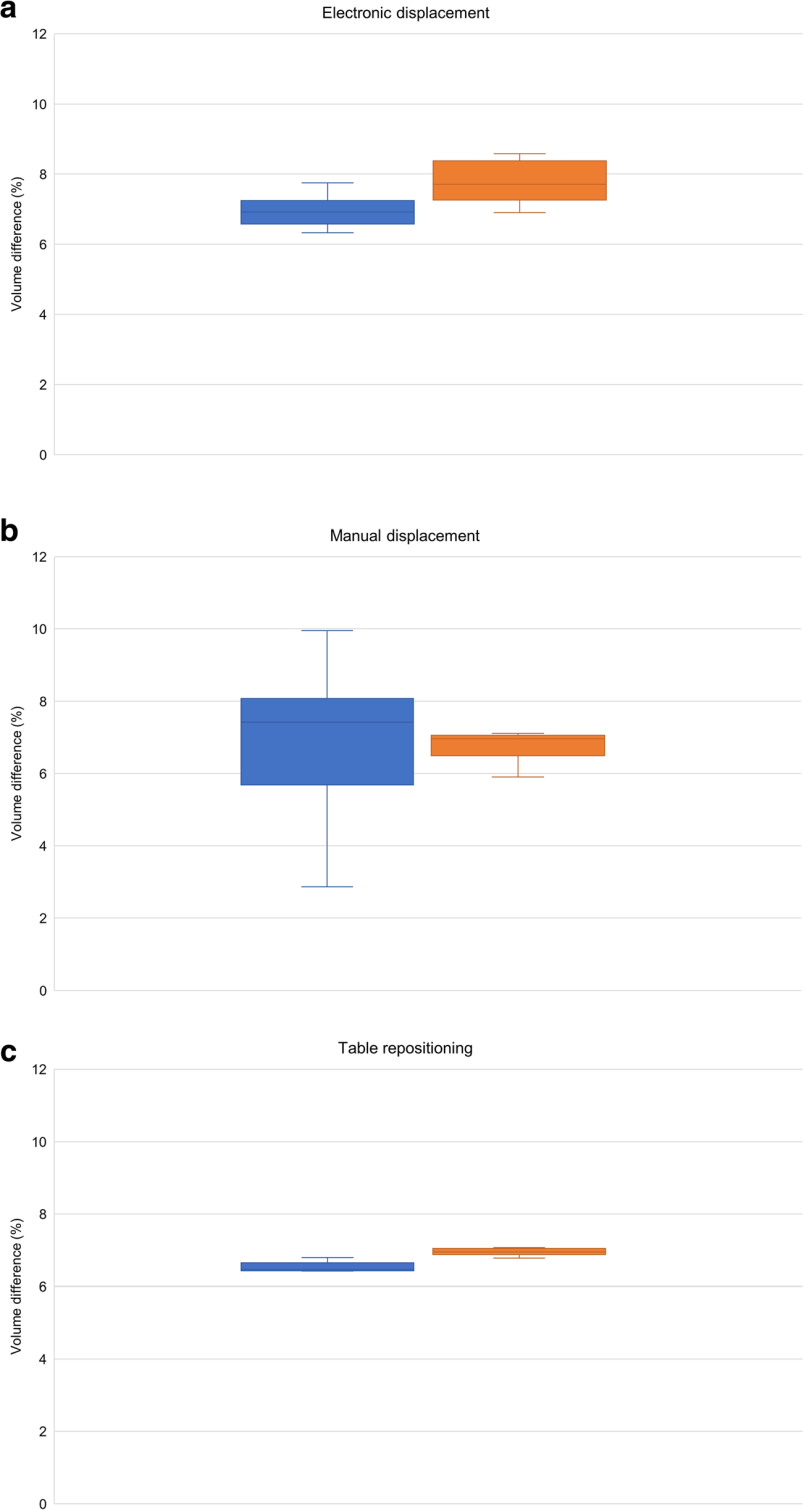

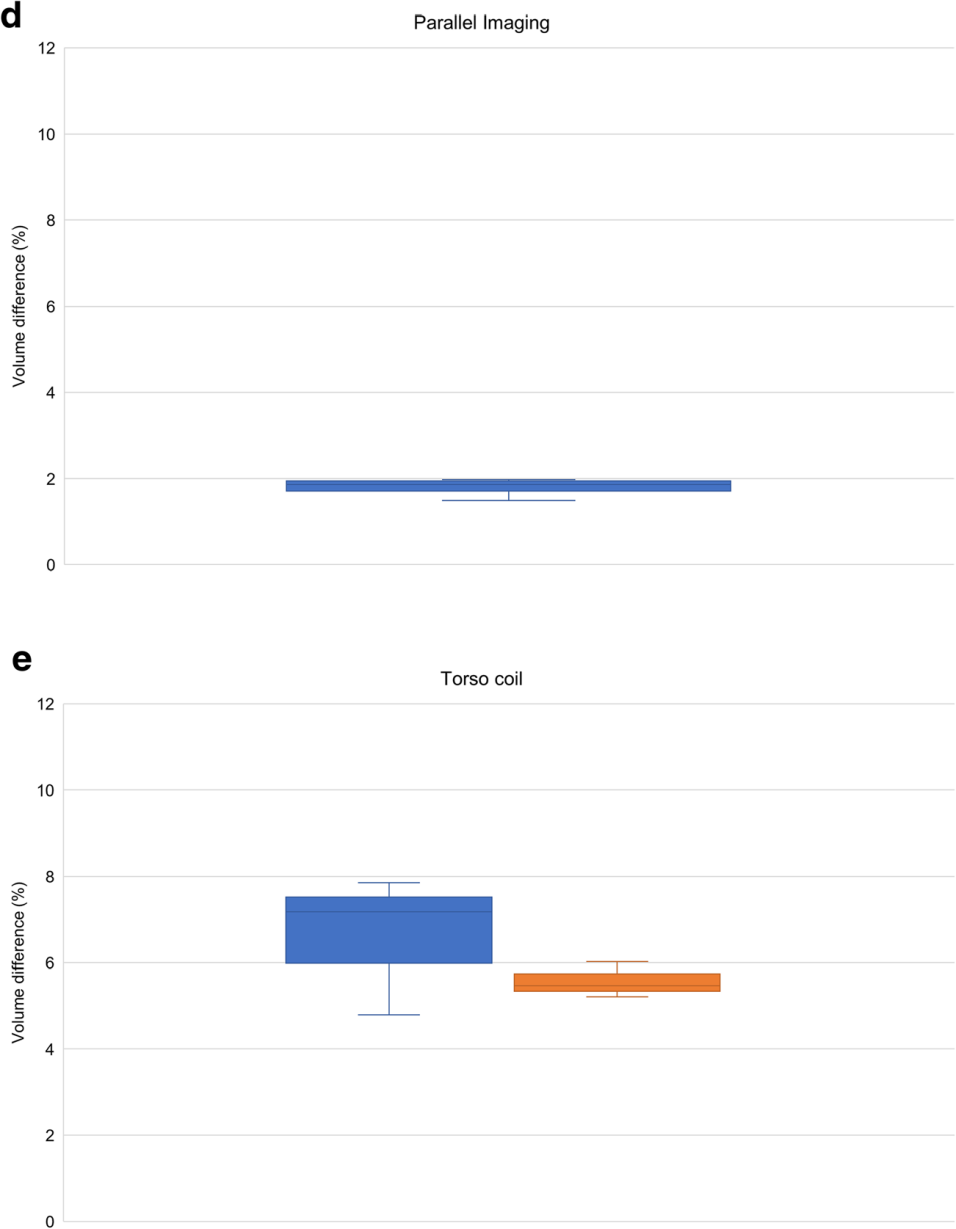
Relative volume difference (%) for **a** electronic displacement of FOV, **b** manual displacement of phantom, **c** table repositioning, **d** parallel imaging, and **e** use of torso coil. The horizontal line through each box indicates the median, rectangular boxes represent the interquartile ranges, and whiskers represent minimum and maximum values. Blue = 2D Gradwarp, orange = 3D Gradwarp

Mean volume of each phantom bottle was 1196.77 ± 77.34 ml measured in CT. Figures 3 and 4 show the effect of GD on volume quantification for different MRI settings. For the reference MRI position with the phantom centered at the isocenter, volume differences were 6.91 ± 0.48 and 6.99 ± 0.22% with 2D and 3D Gradwarp correction, respectively. Electronic displacement of FOV showed volume differences of 6.93 ± 0.57 and 7.81 ± 0.64% with 2D and 3D Gradwarp, respectively. Manual displacement of the phantom showed volume differences of 6.97 ± 8.44 and 6.58 ± 1.42% with 2D and 3D Gradwarp, respectively. Table repositioning showed volume differences of 6.53 ± 0.57 and 6.94 ± 0.25% with 2D and 3D Gradwarp, respectively. Parallel imaging showed a volume difference of 1.80 ± 0.50% with 3D Gradwarp. However, bottles were not completely imaged with this acquisition due to the automatic FOV setting of the scanner, causing an underestimation of the true volume. Imaging using torso coil showed volume differences of 6.61 ± 7.10 and 5.56 ± 1.16% with 2D and 3D Gradwarp, respectively.

**Fig. 5:**
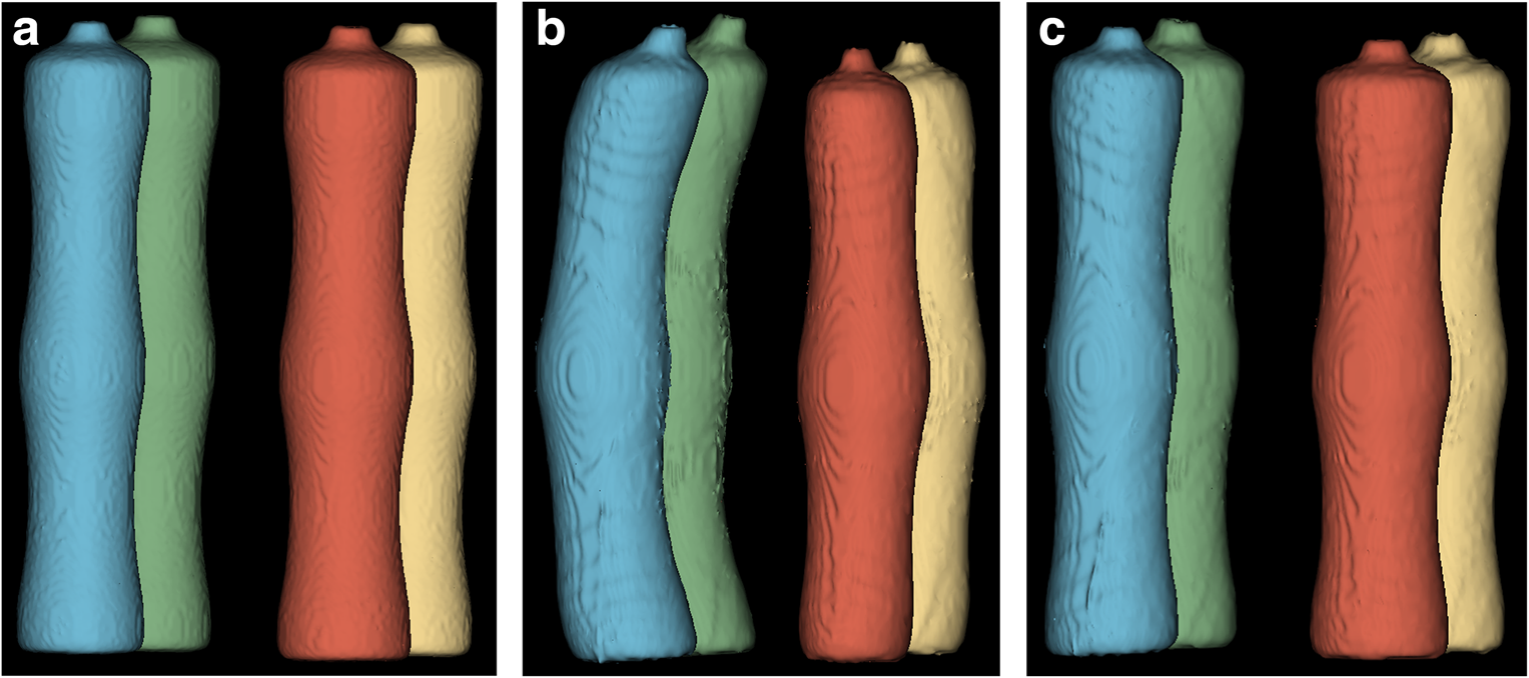
Images illustrate the effect of 2D and 3D Gradwarp. **a** CT reference image, **b** MR image with 2D Gradwarp, **c** MR image with 3D Gradwarp. MR images were obtained with phantom distanced 5 cm to the right of the scanner isocenter. Bending of bottles on the right side of the phantom (blue and green bottles) were seen when the bottles moved further from the scanner isocenter. With 3D Gradwarp, all bottles appear straight

For electronic displacement, volumes of bottles on the right side of the phantom were consistently larger, independently of FOV positioning. Range of volume differences within the phantom was smaller with 3D Gradwarp than with 2D Gradwarp for all MRI settings. Figure 5 shows the effect of 2D and 3D Gradwarp. Bottles were more distorted, when distanced farther away from the isocenter.

### Intra- and intermethod agreement

Phantom volume measurements with 3D Slicer and AWS showed high intra- and intermethod agreement (ICC = 0.991 and ICC = 0.994, respectively). No significant differences between segmentation tools (*Z* = -0.177, *p* = 0.86) were found. A Wilcoxon signed-ranks test with Bonferroni-adjusted alpha levels of 0.025 indicated that the first segmentations were significantly higher in volume than the second segmentations of the same data using 3D Slicer (mean difference = 4.48 ± 7.78 ml, *Z* = -5.450, *p* < 0.001). Similarly, the first segmentations had significantly higher volumes than the second segmentations using AWS (mean difference = 6.36 ± 5.54 ml, *Z* = -7.590, *p* < 0.001). Bland–Altman plots showed that measurements with 3D Slicer and AWS differed very little (Fig. E3).

Figure E4 represents the distribution of Dice scores with 2D and 3D Gradwarp. Mean Dice scores were 0.8593 ± 0.0502 and 0.9298 ± 0.0582 with 2D and 3D Gradwarp, respectively. Range of Dice scores for the MRI settings tested is shown in Table 1. High Dice scores were found for MRI settings using torso coil and table repositioning with 3D Gradwarp. The highest Dice score (0.9611) was found with the torso coil centered on the phantom and with 3D Gradwarp.

**Table 1:** Overlapping Dice (0–1) scores between CT and MR images

MRI setting	Dice score	Dice score
	2D Gradwarp	3D Gradwarp
Reference isocenter position	0.8776	0.9554
Electronic displacement	0.8725–0.9532	0.8788–0.9570
Manual displacement	0.7555–0.8906	0.7559–0.9580
Table repositioning	0.8784–0.8805	0.9542–0.9575
Parallel imaging	N/A	0.9234–0.9378
Torso coil	0.8726–0.8879	0.9468–0.9611

Data are ranges (minimum–maximum) of Dice scores

*N/A* not available

### Quantification of GD on patients’ data

Mean end-inspiratory volume difference between 2D and 3D GW_off-line_ scans was -0.91 ± 2.08%. Mean end-inspiratory volume difference between 2D and 3D GW_scanner_ was 5.50 ± 9.62%. Mean end-inspiratory volume difference between 3D GW_scanner_ and 3D GW_off-line_ was 5.90 ± 9.71%. Based on phantom testing, volume difference between 2D and 3D Gradwarp using torso coil was -0.64 ± 5.59%. Therefore, mean volume difference between 3D GW_scanner_ and 3D GW_off-line_ was around 0.27 ± 11.93%.

### Comparison of segmentation methods

A total of 176 segmentations were analyzed for accuracy, reproducibility, and time efficiency, of which 110 were end-inspiratory segmentations and 66 were end-expiratory segmentations.

**Fig. 6:**
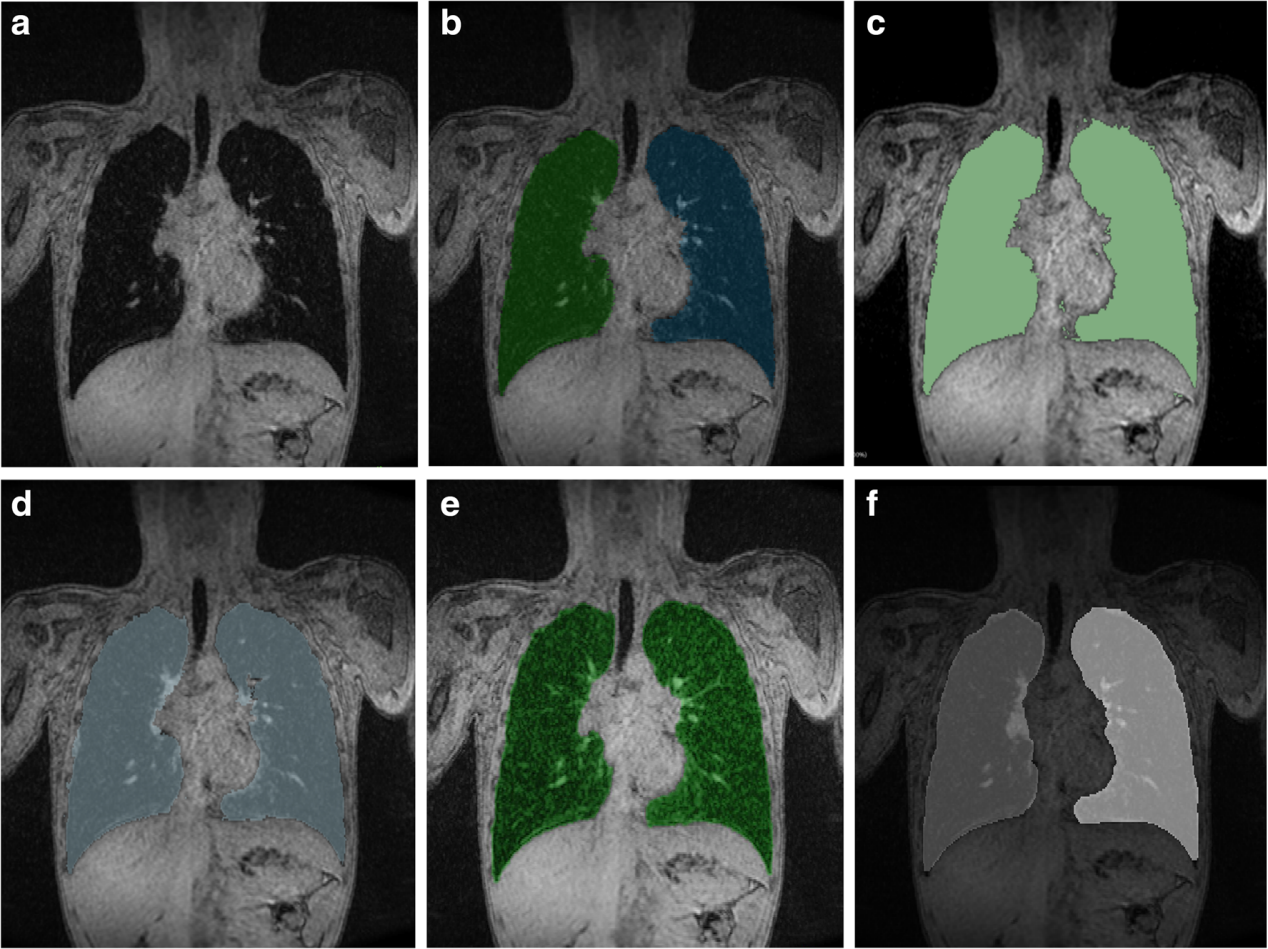
Lung volume segmentations with tested segmentation methods. **a** Exemplary slice with corresponding segmentation results obtained with **b** MS, **c** 3D Slicer, **d** GeoS, **e** Pennati software, and **f** Ivanovska software

Figure 6 shows an example slice and corresponding segmentations with each tested method. Segmentation of mediastinal structures and peripheral lung portions was found to be the source of variation, leading to volume differences between software measurements. Semi-automated and fully automated methods showed similar segmentation errors, namely inclusion of nonlung tissue (i.e., mediastinum) and exclusion of lung tissue at low signal-to-noise regions (i.e., lung’s apices).

### End-inspiratory segmentations

Semi-automated (ICC = 0.988–0.993) and fully automated segmentation results (ICC = 0.971–0.982) showed high agreement with MS (Tables 2 and 3). Results indicated that segmentations with GeoS, Pennati, and Ivanovska methods were similar to MS, with volume differences ranging from 0.59 to 1.37%. One subject was not segmented by Ivanovska’s method due to motion artifacts. 3D Slicer showed a significant difference (*p* < 0.001) with volume differences up to 2.89%. Bland–Altman plots showed good agreement between semi- and fully automated methods and MS (Fig. E5).

**Table 2:** Segmentation time and intermethod agreement (ICC)

Segmentation method	Time (inspiratory)	ICC (inspiratory)	Time (expiratory)	ICC (expiratory)
MS	219 ± 53		149 ± 46	
3D Slicer	47 ± 8	0.988	41 ± 7	0.995
GeoS	12 ± 3	0.993	13 ± 4	0.992
Pennati	2 ± 1	0.982	N/A	N/A
Ivanovska	3 ± 1	0.971	N/A	N/A

Data are ± standard deviation in minutes*.* Semi-automated and fully automated segmentation methods were compared with MS

*N/A* not available, *ICC* intraclass correlation coefficient

**Table 3:** Comparison of end-inspiratory lung volume segmentations of semi-automated and fully automated methods with MS

Segmentation tool	ICC	*N*	Absolute difference (ml)	*p* value*	Relative difference (%)
3D Slicer (semi-automated)	0.988	22	77.99 ± 55.68	< 0.001	2.89
GeoS (semi-automated)	0.993	22	35.19 ± 62.16	0.020	1.30
Pennati (fully automated)	0.982	22	36.92 ± 113.78	0.223	1.37
Ivanovska (fully automated)	0.971	20	15.86 ± 120.83	0.526	0.59

Difference is mean ± standard deviations. Mean inspiratory lung volume obtained with MS was 2702.85 ± 598.51 ml

*ICC* intraclass correlation coefficient, *N* number of subjects

*Calculated with Wilcoxon signed-ranks test with Bonferroni-adjusted alpha levels of 0.01 per comparison

### End-expiratory segmentations

Both semi-automated methods showed high agreement with MS (ICC = 0.992–0.995) (Table 4). They had similar results as MS with mean differences of -1.27 and 1.81% for 3D Slicer and GeoS, respectively. Bland–Altman plots showed a slightly better performance for 3D Slicer than GeoS (Fig. E6).

**Table 4:** Comparison of end-expiratory lung volume segmentations of semi-automated methods with MS

Segmentation tool	ICC	*N*	Absolute difference (ml)	*p* value*	Relative difference (%)
3D Slicer (semi-automated)	0.995	22	-13.63 ± 29.84	0.067	-1.27
GeoS (semi-automated)	0.992	22	19.40 ± 37.65	0.036	1.81

Difference is mean ± standard deviations. Mean expiratory lung volume obtained with MS was 1073.69 ± 334.14 ml

*ICC* intraclass correlation coefficient, *N* number of subjects

*Calculated with Wilcoxon signed-ranks test with Bonferroni-adjusted alpha levels of 0.025 per comparison

### Vital capacity

Spirometry data simultaneously obtained during the MRI scan were available for 8 out of 11 subjects, of which 4 showed large performance variability (Table 5). Association between VC_MRI_ and VC_SPIROMETER_ was similar but not significant for MS (*r* = 0.444, *p* = 0.271), GeoS (*r* = 0.440, *p* = 0.275), and 3D Slicer (*r* = 0.423, *p* = 0.297).

**Table 5:** Spirometry data

Subject	Vital capacity (ml)			
	Spirometry	MS	GeoS	3D Slicer
1	N/A	2310	2313	2230
2	2510	1456	1494	1354
3	1970	605	583	489
4	1790	1828	1816	1752
5	N/A	1852	1888	1784
6	2350	2301	2227	2253
7	N/A	1713	1694	1584
8	1690	1789	1783	1734
9	1520	1486	1453	1357
10	2660	2556	2458	2392
11	2250	1931	1893	1817

*N/A* not available

### Segmentation time

Segmentation time for each method is shown in Table 2. Time displayed excludes time needed for file conversion, uploading, and saving steps. Manual segmentation time for end-expiratory images was shorter than end-inspiratory images, because of the shorter scan range due to lower volumes in expiration.

Both observers found MS a laborious task, aside from the considerable amount of time required for segmentation. Among the semi-automated methods, GeoS performed faster and was less laborious than 3D Slicer. Fully automated methods took approximately 2 to 3 min and required minimal user interaction.

### Intra- and interobserver agreement

High intraobserver agreements were found for GeoS (ICC = 1.000), 3D Slicer (ICC = 0.999), and MS (ICC = 0.997). Volume differences were 4.81 ml (*p* = 0.455), 7.49 ml (*p* = 0.332), and 11.88 ml (*p* = 0.502), respectively, all not significant. High interobserver agreement was found for GeoS (ICC = 0.994), 3D Slicer (ICC = 0.995), and MS (ICC = 0.989). Volume differences were 92.93 ml (*p* < 0.001), 52.91 ml (*p* = 0.030), and 102.03 ml (*p* = 0.001), respectively. With an adjusted alpha level of 0.017, 3D Slicer showed no significant interobserver difference.

## Discussion

We found that mean volume differences from MRI relative to CT due to GD for an object centered at the isocenter of the scanner were 5.56 ± 1.16 and 6.99 ± 0.22% with torso and body coil, respectively. Moreover, we found high Dice overlapping scores for images with 2D Gradwarp and even higher with 3D Gradwarp. We also compared five segmentation methods with different complexity and interaction possibilities. We found that MRI systemically overestimates volume measurements compared to CT due to GD, with varying volume differences according to MRI setting and patient positioning. The range of volume differences within the phantom was always smaller with 3D than 2D Gradwarp, but mean difference was sometimes higher with 3D Gradwarp. This means that 3D Gradwarp normalized the intraobject volume differences but tended to increase volume overestimation.

A discrepancy was found in the expected GD on volume measurements between electronic and manual displacement settings. Previous studies have shown that the greater the distance from the isocenter, the greater the GD [7, 10]. While this was true for the manual displacement setting, it was not for the electronic displacement. Electronic displacement consistently generated a larger volume for bottles on the right side of the phantom. This may be due to asymmetrical inhomogeneity of the magnetic field.

The MRI system changes the FOV from 500 to 600 mm when 3D Gradwarp was applied. When smaller FOV was used with 2D Gradwarp, some bottles were not completely imaged, causing an underestimation of true volume. This problem can explain the lower volume differences found with 2D than with 3D Gradwarp phantom data, for electronic displacement and table repositioning settings.

Finally, end-inspiratory segmentations with GeoS, Pennati, and Ivanovska methods and end-expiratory segmentations with 3D Slicer and GeoS showed similar volume measurements to manual segmentation. Results from the present study suggest that fully automated methods can be used for end-inspiratory lung volume segmentations of large cohort studies, reducing segmentation time and effort without sacrificing accuracy. To date, no fully automated algorithms for end-expiratory images are available. Up to date, GeoS seems the fastest and most accurate semi-automated segmentation method for lung volume segmentation of end-expiratory images.

We acknowledge some limitations to this study: firstly, the small number of subjects. However, each subject had four lung MRI acquisitions, so 44 acquisitions were obtained to test five software methods. Secondly, many missing lung function data hamper data analysis. VC_MRI_ correlated positively with VC_SPIROMETER_, but analysis of a larger subset is needed to confirm these results. This will be eventually achieved when the entire dataset of Generation R is segmented. Thirdly, only one MRI system was used to acquire the MR images. While this ensured homogeneity in the resulting MR images, this does not reflect the wide range of MRI systems and imaging sequences used in practice. However, our approach can be applied to other vendors and MRI sequences.

## Conclusion

MRI systematically overestimates volume compared to CT due to GD. 3D Gradwarp images yield shapes that are similar to reference CT images, but can also determine larger volume overestimation. The effect of GD on volume measurements for images acquired with specific MR settings or in specific patient positions can be quantified and potentially be predicted and corrected.

Semi-automated and fully automated segmentation methods allow accurate, reproducible, and fast lung volume quantification using MRI. We conclude that chest MRI is a valid radiation-free alternative to CT to assess lung volume in large cohort studies.

## Electronic supplementary material

ESM 1 (DOCX 1799 kb)

## Abbreviations

2D Gradwarp: 2D geometry distortion correction technique
3D Gradwarp: 3D geometry distortion correction technique
3D GW_scanner_: 3D Gradwarp correction performed with the built-in software of the scanner
3D GW_off-line_: 3D Gradwarp correction performed with custom-made offline software
3D Slicer: Threshold painting segmentation method (semi-automated segmentation)
AWS: AW Server (semi-automated segmentation)
CT: Computed tomography
FOV: Field of view
GeoS: Geodesic Image Segmentation (semi-automated segmentation)
GD: Geometric distortion
ICC: Intraclass correlation coefficient
MRI: Magnetic resonance imaging
MS: ITK-SNAP segmentation tool (manual segmentation)
VC: Vital capacity

## References

[1] Barreto MM, Rafful PP, Rodrigues RS (2013). Correlation between computed tomographic and magnetic resonance imaging findings of parenchymal lung diseases. Eur J Radiol.

[2] Kuo W, Ciet P, Tiddens HA, Zhang W, Guillerman RP, van Straten M (2014) Monitoring cystic fibrosis lung disease by computed tomography. Radiation risk in perspective. Am J Respir Crit Care Med 189:1328–1336 10.1164/rccm.201311-2099CI 24697683

[3] Tiddens HA, Stick SM, Davis S (2014). Multi-modality monitoring of cystic fibrosis lung disease: the role of chest computed tomography. Paediatr Respir Rev.

[4] van Rikxoort EM, van Ginneken B (2013). Automated segmentation of pulmonary structures in thoracic computed tomography scans: a review. Phys Med Biol.

[5] Mansoor A, Bagci U, Foster B (2015). Segmentation and image analysis of abnormal lungs at CT: current approaches, challenges, and future trends. Radiographics.

[6] Tiddens HA, Rosenow T (2014). What did we learn from two decades of chest computed tomography in cystic fibrosis?. Pediatr Radiol.

[7] Walker A, Liney G, Metcalfe P, Holloway L (2014). MRI distortion: considerations for MRI based radiotherapy treatment planning. Australas Phys Eng Sci Med.

[8] Langlois S, Desvignes M, Constans JM, Revenu M (1999). MRI geometric distortion: a simple approach to correcting the effects of non-linear gradient fields. J Magn Reson Imaging.

[9] Karger CP, Höss A, Bendl R, Canda V, Schad L (2006) Accuracy of device-specific 2D and 3D image distortion correction algorithms for magnetic resonance imaging of the head provided by a manufacturer. Phys Med Biol 51:N253–N261 10.1088/0031-9155/51/12/N04 16757858

[10] Torfeh T, Hammoud R, McGarry M, Al-Hammadi N, Perkins G (2015) Development and validation of a novel large field of view phantom and a software module for the quality assurance of geometric distortion in magnetic resonance imaging. Magn Reson Imaging 33:939–949 10.1016/j.mri.2015.04.003 25882440

[11] Stanescu T, Jans HS, Wachowicz K, Fallone BG (2010). Investigation of a 3D system distortion correction method for MR images. J Appl Clin Med Phys.

[12] Donato F Jr, Costa DN, Yuan Q, Rofsky NM, Lenkinski RE, Pedrosa I (2014) Geometric distortion in diffusion-weighted MR imaging of the prostate-contributing factors and strategies for improvement. Acad Radiol 21:817–823 10.1016/j.acra.2014.02.001 PMC4478612 24709379

[13] Petersch B, Bogner J, Fransson A, Lorang T, Pötter R (2004) Effects of geometric distortion in 0.2T MRI on radiotherapy treatment planning of prostate cancer. Radiother Oncol 71:55–64 10.1016/j.radonc.2003.12.012 15066296

[14] Sumanaweera T, Glover G, Song S, Adler J, Napel S (1994) Quantifying MRI geometric distortion in tissue. Magn Reson Med 31:40–47 10.1002/mrm.1910310106 8121267

[15] Deeley MA, Chen A, Datteri R (2011). Comparison of manual and automatic segmentation methods for brain structures in the presence of space-occupying lesions: a multi-expert study. Phys Med Biol.

[16] Hong C, Lee DH, Han BS (2014). Characteristics of geometric distortion correction with increasing field-of-view in open-configuration MRI. Magn Reson Imaging.

[17] Baldwin LN, Wachowicz K, Thomas SD, Rivest R, Fallone BG (2007) Characterization, prediction, and correction of geometric distortion in 3 T MR images. Med Phys 34:388–399 10.1118/1.2402331 17388155

[18] Kohlmann P, Strehlow J, Jobst B (2015). Automatic lung segmentation method for MRI-based lung perfusion studies of patients with chronic obstructive pulmonary disease. Int J Comput Assist Radiol Surg.

[19] Lui JK, LaPrad AS, Parameswaran H, Sun Y, Albert MS, Lutchen KR (2013) Semiautomatic segmentation of ventilated airspaces in healthy and asthmatic subjects using hyperpolarized 3He MRI. Comput Math Methods Med 2013:624683 10.1155/2013/624683 PMC3626384 23606904

[20] Zhuo J, Gullapalli RP (2006). AAPM/RSNA physics tutorial for residents: MR artifacts, safety, and quality control. Radiographics.

[21] Kruithof CJ, Kooijman MN, van Duijn CM (2014). The generation R study: biobank update 2015. Eur J Epidemiol.

[22] Kooijman MN, Kruithof CJ, van Duijn CM (2016). The generation R study: design and cohort update 2017. Eur J Epidemiol.

[23] Yushkevich PA, Piven J, Hazlett HC (2006). User-guided 3D active contour segmentation of anatomical structures: significantly improved efficiency and reliability. Neuroimage.

[24] Fedorov A, Beichel R, Kalpathy-Cramer J (2012). 3D Slicer as an image computing platform for the Quantitative Imaging Network. Magn Reson Imaging.

[25] Criminisi A, Sharp T, Blake A (2008) GeoS: geodesic image segmentation. Computer vision - ECCV 2008, Pt I, proceedings. Springer, Berlin, pp 99–112

[26] Ivanovska T, Hegenscheid K, Laqua R (2012). A fast and accurate automatic lung segmentation and volumetry method for MR data used in epidemiological studies. Comput Med Imaging Graph.

[27] Pennati F, Quirk JD, Yablonskiy DA, Castro M, Aliverti A, Woods JC (2014) Assessment of regional lung function with multivolume (1)H MR imaging in health and obstructive lung disease: comparison with (3)He MR imaging. Radiology 273:580–590 10.1148/radiol.14132470 PMC4334302 24937692

[28] Zou KH, Warfield SK, Bharatha A (2004). Statistical validation of image segmentation quality based on a spatial overlap index. Acad Radiol.

[29] Ivanovska T, Ciet P, Perez-Rovira A et al (2017) Fully automated lung volume assessment from MRI in a population-based child cohort study. Proceedings of the 12th International Joint Conference on Computer Vision, Imaging and Computer Graphics Theory and Applications - Volume 6: VISAPP. 10.5220/0006075300530058

