# Supplementary material for: Technical challenges of quantitative chest MRI data analysis in a large cohort pediatric study
Source: Eur Radiol. 2018 Dec 5;29(6):2770–82. doi: 10.1007/s00330-018-5863-7 (PMC6510873; doi:10.1007/s00330-018-5863-7)
Supplement: Supplementary file 1 — (DOCX 1799 kb) [file 330_2018_5863_MOESM1_ESM.docx]

**Electronic supplementary material**

**Background of the study**

The Generation R Study is a prospective population-based cohort study among 9,778 mothers and their children. Measurements in the prenatal phase of the study were conducted in close collaboration with midwives and hospitals, and included physical examinations, questionnaires, fetal ultrasound examinations and biological samples. In the preschool period, measurements were conducted in a dedicated research facility at our institution. Data collection in this phase included home visits, physical examinations, questionnaires and hands on assessments and observations in subgroups. At the age of 5, children were invited to the research facility to participate in hands on measurements, behavioral observations and biological sample collections. Children 9 years of age were invited to the research center where beforementioned measurements were continued. General follow-up rate until the age of 10 years was around 80%. Baseline characteristics are described in detail previously [[1](#_ENREF_1); [2](#_ENREF_2)].

**Scan parameters**

*Phantom data*

MR images were acquired on a 3 Tesla MRI system (Discovery MR750w, GE Medical Systems, software DV 24.0) with the following parameters: 3D SPGR, slice thickness 2 mm, FOV 500 mm, flip angle 10°, matrix 250x250, TR 1.656 ms, TE 0.744 ms, torso coil 32 channels.

A CT image data set was acquired using a Siemens PET/CT Biograph mCT with following parameters: slice thickness 1 mm, 360 mA, 120 kVp, pitch 0.60; and with a single source Siemens CT scanner (SOMATOM Definition AS+) with following parameters: slice thickness 1 mm, 300 mA, 140 kVp, pitch 0.60. CT data is known to be distortion free and acts as a gold standard [[3](#_ENREF_3); [4](#_ENREF_4)]. While a description of the geometry of the phantom was provided by the manufacturer, CT data permits the verification of the geometry of this particular phantom, including any manufacturing or structural variances. CT derived volume measurements were used as gold standard and compared with MRI volume measurements.

*Subjects’ data*

Generation R MRI data were acquired on the same MRI system with the following scan parameters: 3D SPGR, slice thickness 2 mm, FOV 400 mm, phase FOV=0.6, flip angle 2°, matrix 200 x 200, TR 1.623 ms, TE 0.724 ms, NEX=0.75 and ARC=5 (phase=2 and slice=2.5), torso coil 32 channels. Two breath-hold end-inspiratory and two breath-hold end-expiratory acquisitions were acquired using an MRI-compatible spirometer (Masterscope Carefusion). Image acquisition was triggered at total lung capacity and at residual volume by a respiratory physiologist.

All MR images, with exception of parallel imaging scans of the phantom, were corrected for GD in both two (2D Gradwarp @ General Electric) and three dimensions (3D Gradwarp @ General Electric). 3D Gradwarp option was not available on the MRI scanner when the Generation R study began. This option became available when the scanner was upgraded to software version (V24) in September 2015. At that time, almost two-thirds of the cohort had already been acquired. For this reason, it was decided to perform 3D Gradwarp correction using an offline software (3D GW_off-line_) provided by General Electrics. To ensure that this offline correction match that of the built-in 3D-Gradwarp software of the scanner (3D GW_scanner_), we compared lung volumes segmentation performed on the end-inspiratory images using the automated software method of Pennati. The dataset of 11 subjects had one end-inspiratory scan without 3D Gradwarp correction (2D GW) and one end-inspiratory scan with 3D correction performed with 3D GW_scanner_. The off-line correction (3D GW_off-line_) could be therefore performed in the first acquisition only. Although the data were spirometry gated, the first and second inspiratory scan are two independent inspiratory maneuvers, which could be slightly different in volume. To overcome this possible source of error, we compared the mean differences between 2D GW-3D GW_off-line_ versus 2D GW-3D GW_scanner_ obtained on constant volumes, that is the first inspiratory scan for the 2D GW-3D GW_off-line_ and the phantom (bottles) for 2D GW-3D GW_scanner_. Therefore, we subtracted ((2D GW-3D GW_off-line_)-((2D GW-3D GW_scanner_). To add an estimate of the SD, we have also computed the expected SD of the difference using Gaußian error propagation, obtained with the formula: sqrt([SD1]²+[SD2]²).

**Imaging analysis**

*Segmentation software tools*

Manual segmentation (MS) of lung contour was obtained for each axial slice using a free-hand contouring tool of ITK-SNAP [[5](#_ENREF_5)]. Manual segmentations were used as reference for comparison with the other segmentation methods. The first semi-automated segmentation method was the Threshold Painting tool of 3D Slicer [[6](#_ENREF_6)]. This tool of 3D Slicer allows to paint voxels in each slice within a pre-determined SI threshold range. SI threshold was defined by setting a ROI within lung’s parenchyma and by excluding soft tissue. The second semi-automated method was the Geodesic image Segmentation (GeoS) [[7](#_ENREF_7)]. GeoS performs lung segmentation by means of brush strokes placed inside and outside the ROI. Lung boundaries are then calculated by using 3D geodesic distance transforms. The first fully automated segmentation method was developed by Ivanovska [[8](#_ENREF_8)]. This method is based on K-Means automatic clustering technique to separate lungs from other tissues. Trachea and main bronchi are localized and extracted using a 3D component analysis procedure. The second fully automated method was developed by Pennati et al [[9](#_ENREF_9)]. This method is based on the automatic selection of the optimal threshold that separates low-density tissue (i.e. lung and air surrounding the subject) from the surrounding chest wall. Trachea and main bronchi are reconstructed using a 3D confidence connected region growing.

**References**

1 Kooijman MN, Kruithof CJ, van Duijn CM et al (2016) The Generation R Study: design and cohort update 2017. Eur J Epidemiol 31:1243-1264

2 Kruithof CJ, Kooijman MN, van Duijn CM et al (2014) The Generation R Study: Biobank update 2015. Eur J Epidemiol 29:911-927

3 Baldwin LN, Wachowicz K, Thomas SD et al (2007) Characterization, prediction, and correction of geometric distortion in 3 T MR images. Med Phys 34:388-399

4 Karger CP, Hoss A, Bendl R et al (2006) Accuracy of device-specific 2D and 3D image distortion correction algorithms for magnetic resonance imaging of the head provided by a manufacturer. Phys Med Biol 51:N253-261

5 Yushkevich PA, Piven J, Hazlett HC et al (2006) User-guided 3D active contour segmentation of anatomical structures: significantly improved efficiency and reliability. Neuroimage 31:1116-1128

6 Fedorov A, Beichel R, Kalpathy-Cramer J et al (2012) 3D Slicer as an image computing platform for the Quantitative Imaging Network. Magn Reson Imaging 30:1323-1341

7 Criminisi A, Sharp T, Blake A (2008) GeoS: Geodesic Image Segmentation. Computer Vision - Eccv 2008, Pt I, Proceedings 5302:99-112

8 Ivanovska T, Hegenscheid K, Laqua R et al (2012) A fast and accurate automatic lung segmentation and volumetry method for MR data used in epidemiological studies. Comput Med Imaging Graph 36:281-293

9 Pennati F, Quirk JD, Yablonskiy DA et al (2014) Assessment of regional lung function with multivolume (1)H MR imaging in health and obstructive lung disease: comparison with (3)He MR imaging. Radiology 273:580-590

**Figures**


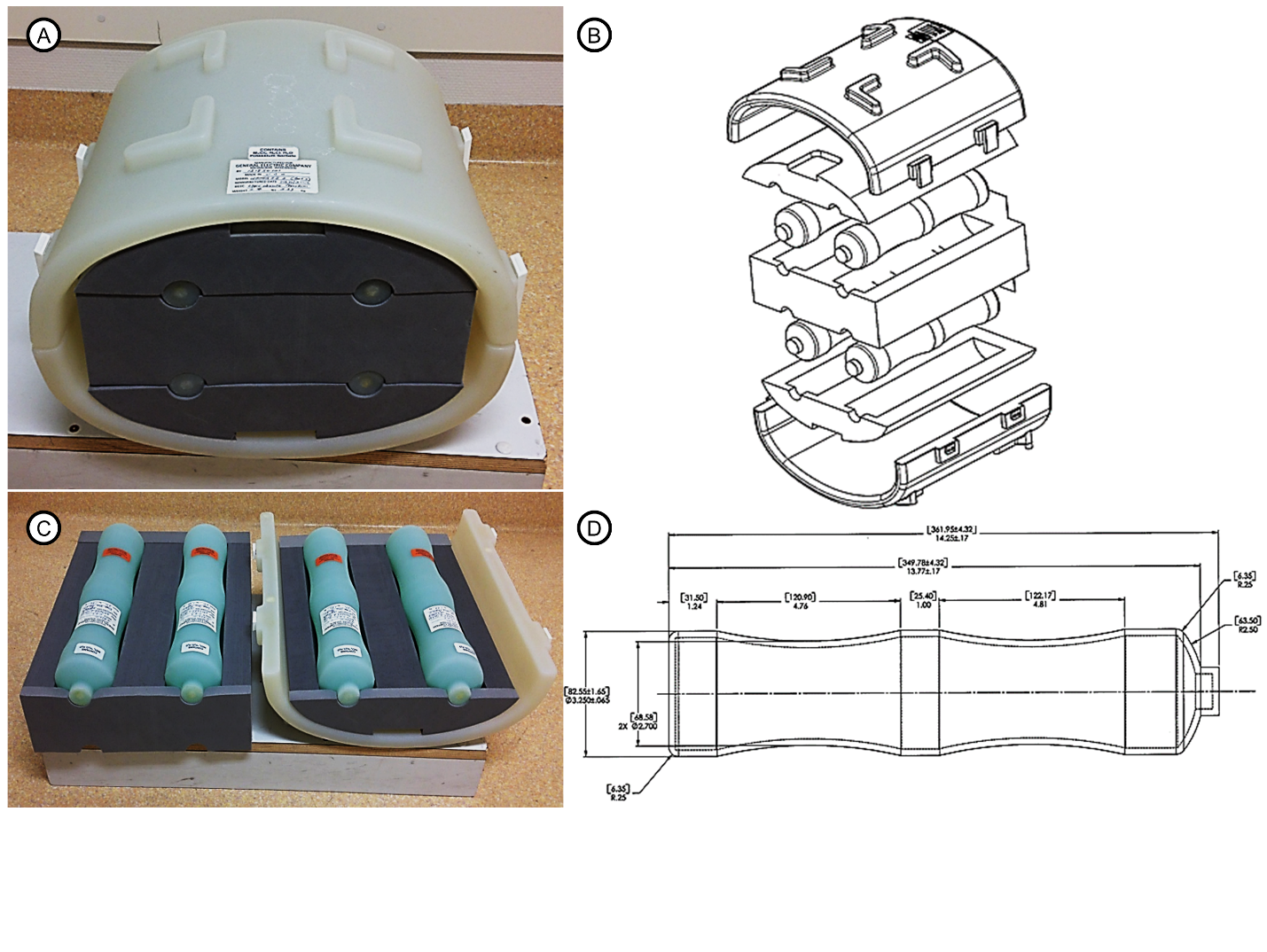


*Figure E1. General Electric body phantom (GE, Healthcare). A) Assembled Body Phantom, B) Assembling scheme, C) Disassembled Phantom with the four bottles of Potassium Sorbate and C) Dimensions of each bottle.*

*Figure E2. Flowchart of tested segmentation methods. Each subject (n=11) underwent two end-inspiratory and two end-expiratory acquisitions. Segmentation with one manual method (ITK-SNAP), two semi-automated methods (3D Slicer and GeoS), and two fully automated methods (Ivanovska and Pennati) were performed on each acquisition.*


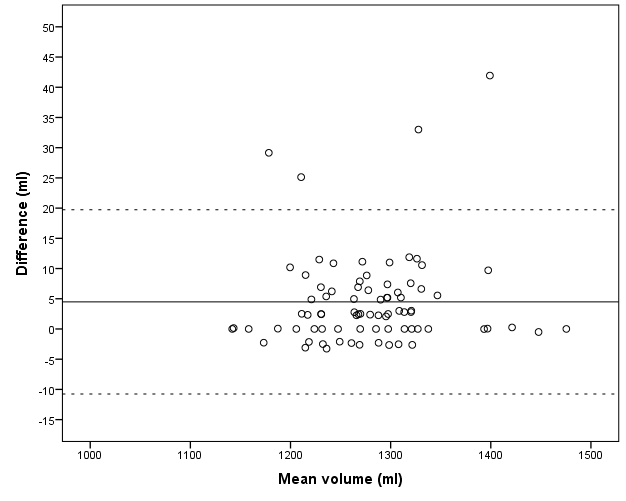


A


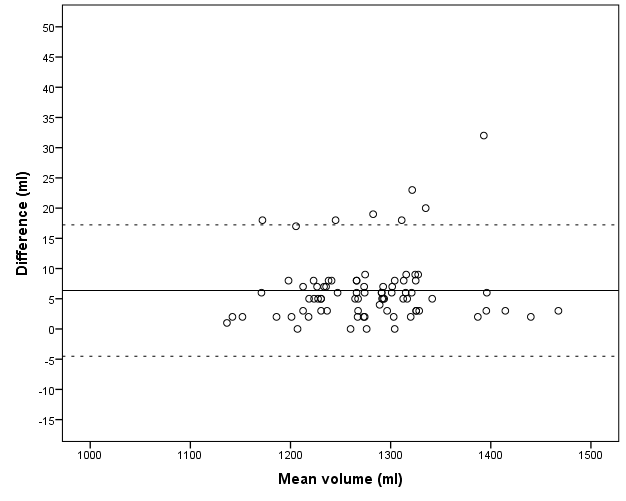


B

*Figure E3. Intra-method differences with Bland Altman plots. A) Bland Altman plot of measurements with 3D Slicer. B) Bland Altman plot of measurements with AW Server. Solid line is the mean difference between first and second segmentations; dotted lines show 95% confidence interval.*


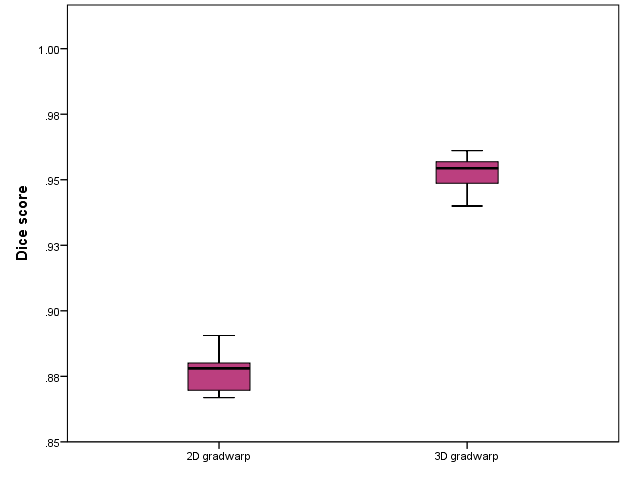
*Figure E4. Dice scores with 2D and 3D Gradwarp as a measure of overlap between MR and CT images. The horizontal line through each box indicates the median of Dice scores, rectangular boxes represents the interquartile ranges, whiskers represents Dice scores within 1.5 times the interquartile range.*


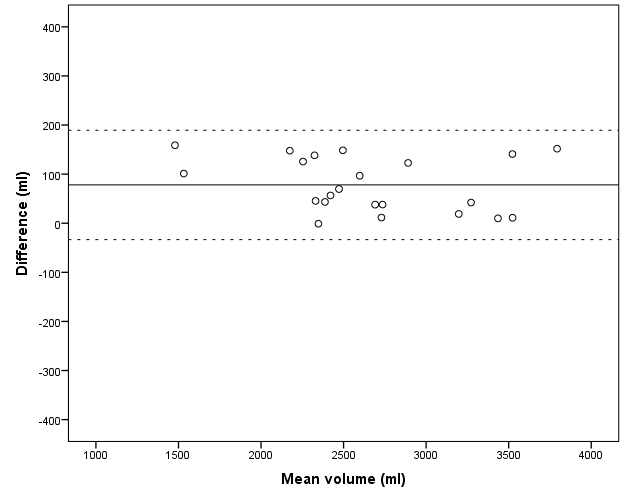


A


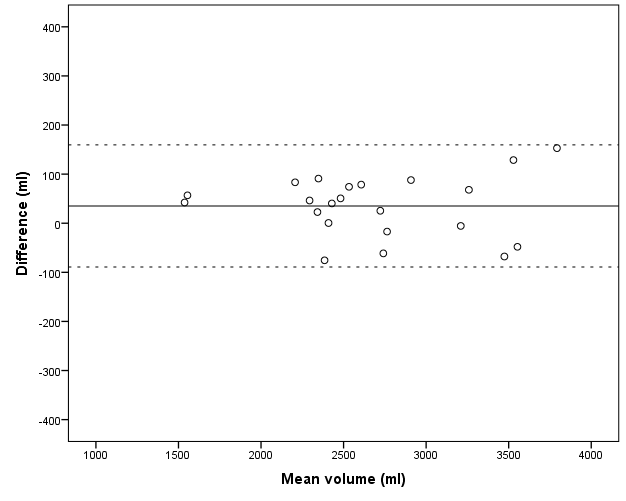


B


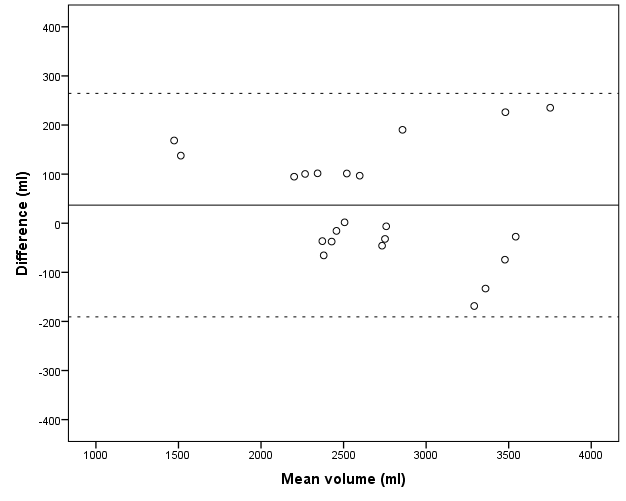


C


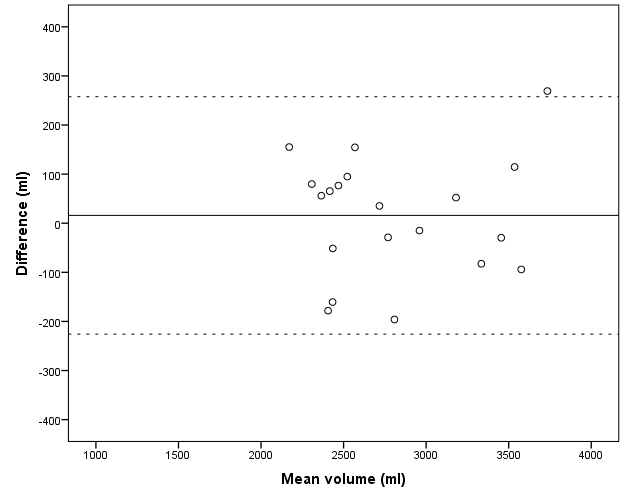


D

*Figure E5. Inter-method differences for segmentation of end-inspiratory images with Bland Altman plots. Solid line is mean difference; dotted lines show 95% confidence interval. Comparison of a) 3D Slicer, b) GeoS, c) Pennati, d) Ivanovska software segmentations with MS.*


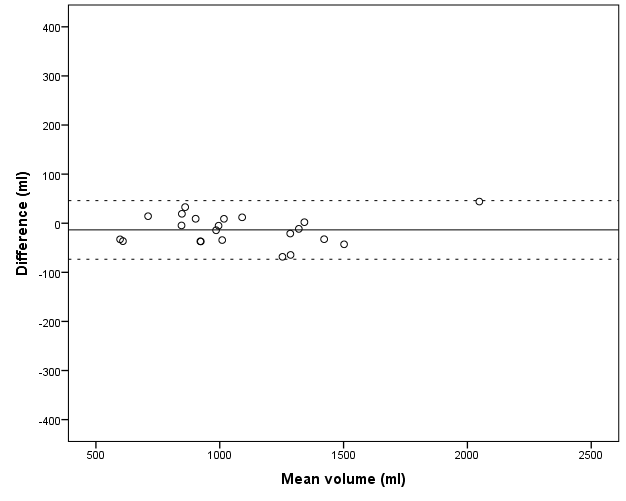


A


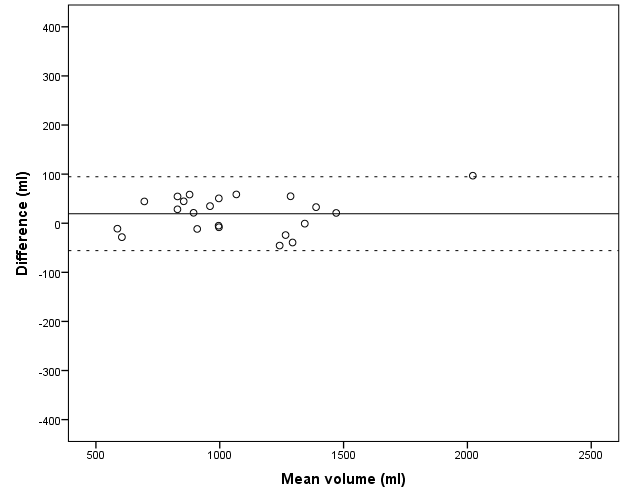
*Figure E6. Inter-method differences for segmentation of end-expiratory images with Bland Altman plots. Solid line is mean difference; dotted lines show 95% confidence interval. Comparison of a) 3D Slicer and b) GeoS segmentations with MS.*

B
